# Molecular characterization of a *Rhodococcus jostii* RHA1 γ-butyrolactone(-like) signalling molecule and its main biosynthesis gene *gblA*

**DOI:** 10.1038/s41598-017-17853-6

**Published:** 2017-12-18

**Authors:** Ana Ceniceros, Lubbert Dijkhuizen, Mirjan Petrusma

**Affiliations:** Microbial Physiology, Groningen Biomolecular Sciences and Biotechnology Institute (GBB), University of Groningen, Nijenborgh 7, 9747AG Groningen, The Netherlands

## Abstract

*Rhodococcus* genome sequence analysis has revealed a surprisingly large (and unexplored) potential for the production of secondary metabolites. Also, putative γ-butyrolactone gene clusters have been identified in some Rhodococci. These signalling molecules are known to regulate secondary metabolism in *Streptomyces*. This work provides evidence for synthesis of a γ-butyrolactone(-like) molecule by Rhodococci (RJB), the first report in the *Rhodococcus* genus. The *Rhodococcus jostii* RHA1 RJB molecule was detected by a reporter system based on the γ-butyrolactone receptor protein (ScbR) of *Streptomyces coelicolor*. This RJB is structurally identical to 6-dehydro SCB2, the predicted precursor of the *S*. *coelicolor* γ-butyrolactone SCB2. The *R*. *jostii* RHA1 key RJB biosynthesis gene was identified (*gblA*): Deletion of *gblA* resulted in complete loss of RJB synthesis whereas higher RJB levels were detected when *gblA* was overexpressed. Interaction of the RJB molecule with ScbR indicates that communication may occur between these two Actinomycete genera in their natural habitat. Furthermore, RJB may provide a highly relevant tool for awakening cryptic secondary metabolic gene clusters in Rhodococci. This study provides preliminary evidence that *R*. *jostii* RHA1 indeed synthesizes diffusible molecules with antimicrobial activity, but a possible role for RJB in this remains to be established.

## Introduction

*Rhodococcus* is a genus of aerobic, acid resistant, non-sporulating, Gram-positive soil bacteria (family Nocardiaceae, order Actinomycetales), which contain mycolic acids in their cell walls^1^. This genus is well-known for its catabolic versatility^2–5^, but little is known about its secondary metabolism. Computational analysis has shown that this genus has a great potential for synthesis of secondary metabolites^4,6–10^. Analysis of several Actinomycete genomes, including 4 strains of the genus *Rhodococcus*, *R*. *jostii* RHA1*, R*. *equi* 103 S*, R*. *opacus* B4 and *R*. *erythropolis* PR4, uncovered a relatively high percentage of genes encoding non-ribosomal peptide synthetases (NRPS) in Rhodococci. Also, conserved γ-butyrolactone biosynthesis gene clusters were identified in these Rhodococci^7^. The physiological roles of γ-butyrolactones have been extensively studied in members of the genus *Streptomyces* only, although their putative biosynthetic genes also appear to be present in other Actinomycete genera^11^. These signalling molecules are known to participate in the regulation of secondary metabolism and to induce a range of physiological responses^12–15^. γ-Butyrolactones bind to one or more receptor proteins, which belong to the TetR family of transcriptional regulators. Several of these receptor proteins have been described in the genus *Streptomyces* to be involved in controlling expression of secondary metabolite gene clusters^15–19^. The γ-butyrolactone TetR receptors bind to DNA, thus blocking the expression of the target genes. Binding of γ-butyrolactone ligands to the TetR regulators induces a change in the receptor protein conformation. The receptor therefore, cannot bind to the DNA anymore influencing the expression of the targeted genes. For example, in *Streptomyces coelicolor* the γ-butyrolactones receptor protein (ScbR) directly regulates the coelimycin gene cluster. Lack of γ-butyrolactones inhibits the production of coelimycin^20^. In *Streptomyces griseus* γ-butyrolactones are known to regulate streptomycin production and morphological differentiation^15^.

**Figure 1:**
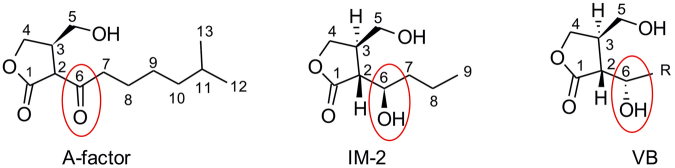
Illustration of the three γ-butyrolactone structural types described to date, differing in group and conformation at the C6 position. A-factor was first described in *Streptomyces griseus* ^15^; IM-2 molecules were first identified in *Streptomyces lavendulae* ^24^; VB molecules were first reported for *Streptomyces venezuelae* ^22^. Adapted from Martin-Sanchez *et al*.^27^.

Three main groups of γ-butyrolactones have been described to date, classified according to their structures: A-factor type, which contains a keto group in the carbon 6 of the molecule; IM-2, with a hydroxyl group in the same carbon in the R configuration; and VB type, also with a hydroxyl group in the same carbon but in an S configuration^21–25^ (Fig. 1). Within each group there is further diversity depending on the structure of the acyl chain connected to carbon 6.

The enzymes involved in the biosynthesis of γ-butyrolactones have been described and partially characterized in several *Streptomyces* strains. They have been named according to the species that employ them or the compound that is regulated by these molecules^15–17,19,26,27^. For unification purposes, we have renamed these enzymes so that they can be applied to any butanolide system in any strain by employing the nomenclature Gbl (**g**amma-**b**utyro**l**actone) (Table 1).

**Table 1:** Proposed unified nomenclature of described GBL biosynthesis enzymes.

Strain	GblA	GblB	GglC	GblR	GblE	Reference
*S*. *griseus*	AfsA	—	—	ArpA	—	15
*S*. *coelicolor*	ScbA	ScbB	ScbC	ScbR	—	27
*S*. *venezuelae*	JadW1	Jadw3	—	JadR2	JadW2	47
*R*. *jostii* RHA1	RHA1_RS22510	—	—	RHA1_RS22505	RHA1_RS22500	This work

**Figure 2:**
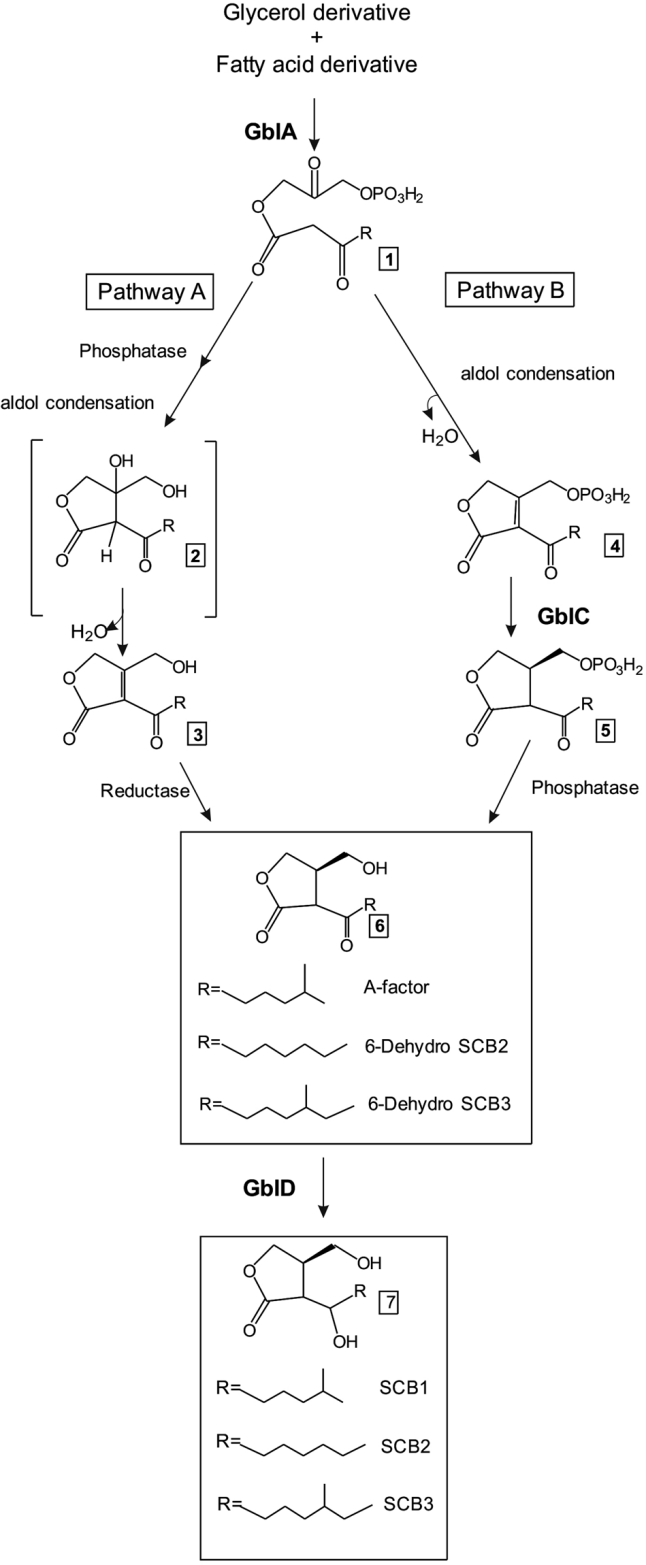
Predicted biosynthetic pathway of γ-butyrolactones in *Streptom*yces species (adapted from^15,27,48^) The generic names given to the enzymes in this work are shown at the (putative) steps that they catalyse. A-factor is the γ-butyrolactone from *S*. *griseus*. SCBs (*Streptomyces Coelicolor* Butyrolactones), γ-butyrolactones described in *Streptomyces coelicolor*.

GblA (**g**amma-**b**utyro**l**actone biosynthesis **A**) catalyses the first step of the biosynthesis by condensing a glycerol derivative with a fatty acid derivative (Compound 1, Fig. 2). This enzyme was first described in *S*. *griseus* where it was named AfsA (Fig. 2)^15^. After this step two different pathways have been predicted^15^. Reactions in Pathway A are believed to be catalysed by non-specific enzymes. Pathway B includes a reductase GblC (**g**amma-**b**utyro**l**actone biosynthesis **C**), named BprA in *S*. *griseus* (Fig. 2)^15^ and a phosphatase that is thought to be a non-pathway-specific phosphatase. GblC is predicted to reduce the double bond in carbons 3 and 2 in the lactone ring (conversion of compound 4 into 5). In some species, there is also a short chain dehydrogenase (GblD, **g**amma-**b**utyro**l**actone biosynthesis **Dehydrogenase**) that reduces the keto group in carbon 6 of 6-dehydro-γ-butyrolactone forms (compound 6 to compound 7 in Fig. 2).

The butanolide system has only been studied as a quorum-sensing system in *Streptomyces* but it has not been explored whether it can have a role in interspecies communication; there is evidence that other genera also have this system^11,28^. In this work, we identified a γ-butyrolactone(-like) molecule in *R*. *jostii* RHA1 (RJB), the first time that such a molecule has been identified in the genus *Rhodococcus*. A *R*. *jostii* RHA1 *gblA* deletion mutant did not induce the growth of a γ-butyrolactone reporter strain^25^, indicating that the *gblA* gene is essential for synthesis. Moreover, an overproduction of RJB was observed when *gblA* was overexpressed in *R*. *jostii* RHA1. LC-MS analysis of extracts from the *R*. *jostii* RHA1 WT strain and the derived *gblA* deletion and the *gblA* overexpression strains indicated that the RJB molecule synthesized has the same structure as 1) 6-dehydro SCB2, a stereoisomer of A-factor, the known γ-butyrolactone from *S*. *griseus* and 2) a predicted precursor of SCB2 (Streptomyces Coelicolor Butyrolactone 2) (Figs 1, 2), a known γ-butyrolactone from *S*. *coelicolor* ^27^.

## Materials and Methods

### Strains and growth conditions

All strains used in this study are described in Table 2 and the media used in Supplementary Table S1. *Rhodococcus* was grown at 30 °C. Luria-Bertani agar from Sigma-Aldrich was used as a standard medium. Appropriate antibiotics were used at the following concentrations: apramycin 50 μg/ml, kanamycin (Km) 200 μg/ml.

**Table 2:** Microbial strains used in this work.

Strain	Details	Reference
*Rhodococcus jostii* RHA1	Wild type	4
RHA1-OE	*R*. *jostii* RHA1 + pRM4-*gblA*	This work
RHA1-Δ*gblA*	*R*. *jostii* RHA1Δ*gblA*	This work
RHA1-C	*R*. *jostii* RHA1Δ*gblA* + pRM4-*gblA*	This work
*S*. *coelicolor* LW16/pTE134	γ-Butyrolactone reporter strain. *S*. *coelicolor* M145 Δ*scbA*Δ*scbR* (LW16) containing the construct pTE134 (*scbR* and Km resistance gene under the control of a γ-butyrolactone inducible promoter (*cpkOp*)	33
*S*. *coelicolor* M145	Wild type strain of *S*. *coelicolor*	49
*E*. *coli* DH5α^TM^	Cloning strain. F– Φ80*lac*ZΔM15 Δ(*lac*ZYA*-arg*F) U169 *rec*A1 *end*A1 *hsd*R17 (rK−, mK + ) *pho*A *sup*E44 λ– *thi*-1 *gyr*A96 *rel*A1	Invitrogen
*Aspergillus niger* N402	Bioactivity reporter strain. Wild type.	50
*Micrococcus luteus* ATCC9341/ *Kocuria rhizophila*	Bioactivity reporter strain. Wild type.	
*Bacillus subtilis* ATCC6633	Bioactivity reporter strain. Wild type.	
*E*. *coli* JM101	Bioactivity reporter strain F′ traD36 *proA* + *B* + *lacIq* Δ(*lacZ*)M15/Δ*(lac-proAB*) *glnV thi*	NEB
*Mycobacterium smegmatis* MC2 155	Bioactivity reporter strain. Wild type.	

### Phenotypic characterization of the different strains

R. *jostii* RHA1 WT, RHA1*-*Δ*gblA* and RHA1*-*OE were grown on different solid media to check for phenotypic differences. Growth, pigmentation and antibiotic activity against *Aspergillus niger, Mycobacterium smegmatis, Escherichia coli, Bacillus subtilis* and *Micrococcus luteus* on solid media were monitored by visual observation. *M*. *smegma*tis and *M*. *luteus* were grown at 30 °C and *E*. *coli* and *B*. *subtilis* were grown at 37 °C before inoculating the media for the bioassay. Afterwards plates were kept at 30 °C. Cell shapes were studied under a Zeiss Axioskop 2 phase contrast microscope. The media used for antibiotic production tests were Trypton Soya Agar (TSA), Difco Nutrient Agar (DNA), Luria Broth Agar (LBA), Starch Casein Agar (SCA), Minimum Salt Medium (MSM) nitrogen deficient and complimented with casamino acids, Supplemented Minimum Medium Solid (SMMS) and low pH SMMS: in this case pH was not adjusted after mixing components (Supplementary Table S1). *R*. *jostii* RHA1 transformants were grown for 4 days before plating the bioassay test strains (Table 2) next to the *Rhodococcus* colonies. Growth of the antimicrobial bioassay test strains was followed over time and scored after 4 days.

### Bioinformatic analysis

The following complete genome sequences of Rhodococci were obtained from GenBank (http://www.ncbi.nlm.nih.gov/genbank/): *Rhodococcus jostii* RHA1, *Rhodococcus aetherivorans, Rhodococcus equi* 103 S*, Rhodococcus equi* ATCC 33707, *Rhodococcus erythropolis* CCM2595, *Rhodococcus erythropolis* PR4, *Rhodococcus erythropolis* R138, *Rhodococcus opacus* B4, *Rhodococcus opacus* PD630, *Rhodococcus opacus* R7, *Rhodococcus pyridinivorans* SB3094, *Rhodococcus* sp. AD45, *Rhodococcus* sp. B7740, *Rhodococcus* sp. BCP1. AntiSMASH (http://antismash.secondarymetabolites.org/) was used for the detection of γ-butyrolactone biosynthetic gene clusters in these genomes using the ClusterFinder algorithm.

### Deletion mutagenesis

Unmarked gene deletion mutagenesis^29^ was used to delete *gblA*. Primers afsA-del-GTG-FW-XbaI and afsA-del-GTG-Rv-EcoRI and afsA-del-UAG-Fw-PstI and afsA-del-UAG-Rv-XbaI (Table 3) were designed to amplify fragments of 1.5 Kb upstream and downstream of *gblA*, including the first and last ~200 bp from *gblA* to ensure that the surrounding genes were not affected by the deletion. Genomic DNA was used as template. Both fragments were cloned in pK18mobSacB (ATCC® 87097™) between EcoRI and PstI, producing the deletion construct pK18mobSacB-Δ*gblA*. The deletion construct was transformed into *R*. *jostii* RHA1 by electrotransformation. First cross-over colonies were selected for Km resistance and sucrose sensitivity. Second recombination was selected by Km sensitivity and sucrose resistance. Deletion mutants were checked by PCR using genomic DNA with primers outside the 1.5 Kb homologous regions and by sequencing of the resulting product using primers afsA-check-Fw-2 and afsA-check-Rv-1 and afsA-check-Fw-2 and afsA-check-Rv-2 (see Fig. S1 and Table 3).

**Table 3:** Primers used in this work.

Primer	Sequence	Amplicon target
ScbA-jostii-NdeI-F	GCGATACATATGGCGCAAATTTCCCGGCCGAT	*R*. *jostii gblA* gene
ScbA-jostii-BamHI-R	CGCTATGGATCCCTAGCGAGCGCATGCGCTCA	*R*. *jostii gblA* gene
afsA-del-GTG-FW-XbaI	GATTATCTAGAGAAGACCTCGGCCACGGATTG	Upstream region of *R*. *jostii gblA* gene for deletion
afsA-del-GTG-Rv-EcoRI	TACTTGAATTCGGGCTTTCGTGAACGACCTC	Upstream region of *R*. *jostii gblA* gene for deletion
afsA-del-UAG-Rv-XbaI	GATTATCTAGAGACGAGCGAGCCACGATCC	Downstream region of *R*. *jostii gblA* gene for deletion
afsA-del-UAG-Fw-PstI	GTCAACTGCAGGCCGGGCGAGATCGTTCAC	Downstream region of *R*. *jostii gblA* gene for deletion
afsA-del-check-1-Fw	CGACGCCGACTAGCGAGC	Primer annealing outside the 1.5 KB homologous region used for the double recombination to delete *afsA*
afsA-del-check-1-Rv	TGGTCGCGGTTACTGGACAC	Primer annealing outside the 1.5 KB homologous region used for the double recombination to delete *afsA*
afsA-del-Check-2-FW	TGGCAGGCGTGGAACACGTC	Primer annealing outside the 1.5 KB homologous region used for the double recombination to delete *afsA*
afsA-del-check-2-Rv	GTCGTTCGAGCGGACCGTTC	Primer annealing outside the 1.5 KB homologous region used for the double recombination to delete *afsA*
pSET152-CS-Fw	TACCGCATCAGGCGCCATTC	Primer annealing at one side of the insertion in PMR4
pSET152-CS-Rv	TTATGCTTCCGGCTCGTATG	Primer annealing at one side of the insertion in PMR4

### *gblA* complementation and overexpression

Primers ScbA-jostii-NdeI-F and ScbA-jostii-BamHI-R (Table 3) were used to amplify the *R*. *jostii* RHA1 *gblA* gene (969 bp) and clone it into pRM4^30^ under the control of the strong constitutive promoter ermE*(pRM4-*gblA*). This construct was introduced into *R*. *jostii* RHA1 wild type strain and RHA1-Δ*gblA* by electrotransformation obtaining the transformants RHA1-OE and RHA1-C, respectively. Clones were selected by apramycin resistance and checked by PCR with primers pSET152-CS-Fw and pSET152-CS-Rv (Table 3) annealing in pRM4 at both sides of the insert and by sequencing of the resulting products.

### Transformation of *Rhodococcus*

A modified electrotransformation protocol from Arenskotter *et al*.^31^ was used to introduce the different constructs described in this work into *R*. *jostii* RHA1. Strains were grown in 50 ml LB containing 1% w/v of glycine in a 250 ml Erlenmeyer flask at 30 °C and 220 rpm to an OD600 of 0.8–1. Cells were washed twice with 15 ml of chilled deionized water and concentrated to 2.5 ml in 10% glycerol and aliquoted in 400 μl. Subsequently, 100 ng to 1 μg of DNA was added to each 400 μl and the sample was kept on ice for at least 10 min. Cells were pulsed with a Biorad Xcell gene pulser at 1.75 kV, 50 μF and 200 Ω (field strength of 8.75 kV cm^−1^). Ice cold LB was added immediately after the pulse and the cell samples were allowed to recover for 4 h at 30 °C and 220 rpm. Subsequently the cells were plated on selective media.

### γ-Butyrolactone extraction

*Rhodococcus* strains were grown in modified SMM solid (SMMS) medium^32^ (Supplementary Table S1) at 30 °C. Extraction of γ-butyrolactones was performed following the procedure described in Hsiao *et al*.^33^. *R*. *jostii* RHA1 WT and transformant strains derived were grown on modified SMMS^32^ (Table S1). Per strain, 40 standard (90 mm diameter) petri dishes were used. After 4 days of growth at 30 °C, when the strains turned orange due to carotene production, which is an indication of an active secondary metabolism, the agar of each plate was cut into pieces and extracted with ethyl acetate as described in Hsiao *et al*.^33^. Extracts were dried at 30 °C in a rotary evaporator, and then resuspended in 160 μl of methanol per 40 petri dishes.

### GBL specific reporter assay (Kanamycin (Km) assay)

The GBL-specific reporter assay performed in this study were done following the protocol from Hsiao *et al*.^33^. From the extract of each strain, 60 μl were concentrated to 6 μl by evaporation in a Savant DNA SpeedVac and spotted onto a DNA plate (Supplementary Table S1) containing 4.5 µg ml^−1^ of Km and plates were uniformly spread with *S*. *coelicolor* strain LW16/pTE134. *Streptomyces* was plated before adding the extracts. As positive control, 6 μl of a stock solution of 1.5 mg ml^−1^ of chemically synthesized 6-dehydro SCB2 was used. Dried ethyl acetate resuspended in methanol was used as negative control. Results were reproducible with 2–3 biological replicates for each strain.

### Liquid chromatography-Mass spectrometry analysis

For identification of *R*. *jostii* RHA1 γ-butyrolactone-like molecules, HPLC-MS analysis was performed using an Accella1250™ HPLC system coupled with the benchtop ESI-MS Orbitrap Exactive™ (Thermo Fisher Scientific, San Jose, CA). A Reversed Phase C18 (Shim Pack Shimadzu XR-ODS 3 × 75 mm) column was used and a gradient from 2% to 95% of acetonitrile:water (0.1% Formic Acid) as follows: 2 min 2% acetonitrile, 2–10 min gradient to 95% acetonitrile, 1 min 95% acetonitrile. To separate further the peaks from A-factor and 6-dehydro SCB2, a gradient from 2% to 80% acetonitrile was applied to the separation: 2 min 2% acetonitrile, 2–25 min in 2–80% acetonitrile, 1 min 80% acetonitrile. Data was analysed using Xcalibur software from Thermo Scientific. LC-MS analysis was performed with 2–4 biological replicates per strain.

### Synthesis of γ-butyrolactone standards

Synthetic SCB1, SCB2, A-factor and 2-dehydro SCB2 γ-butyrolactones used in this study were chemically synthesized as described by Martin-Sanchez^27^.

### Analysis of the interactions between *Streptomyces coelicolor* and *R*. *jostii* RHA1

*R*. *jostii* RHA1 wild type, strains RHA1-OE and RHA1-Δ*gblA* were plated onto modified minimum salt media (MSM) containing casamino acids instead of NH_4_NO_3_ (see Supplementary Table S1). After 4 days, *S*. *coelicolor* M145 was plated next to the patches of the *R*. *jostii* strains. Following a further incubation for 18 h, the *S*. *coelicolor* growth stage and production of coloured antibiotics^20^ was checked every 2 h; after 24 h of incubation these parameters were checked once a day for a week. Experiments were performed in 5 independent replicas.

### Data availability statement

All data generated or analysed during this study are included in this published article (and its Supplementary Information files).

## Results

### γ-Butyrolactone gene clusters in Rhodococci

**Figure 3:**
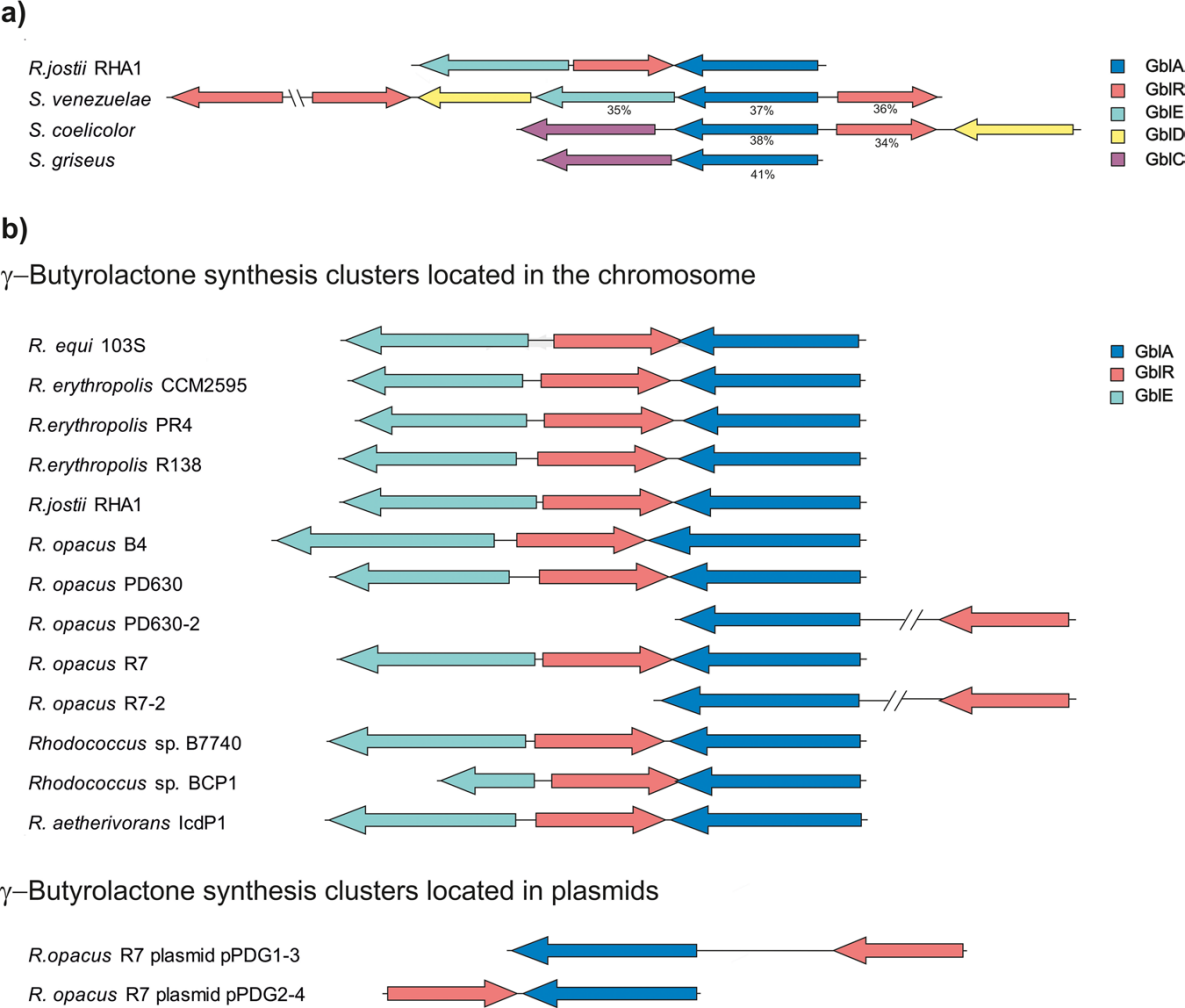
Predicted γ-butyrolactone gene clusters in different strains. (**a**) Organization of the predicted γ-butyrolactone gene cluster of *Rhodococcus jostii* RHA1 compared to that of the known clusters in different *Streptomyces* strains. AA identity of the *R*. *jostii* RHA1 enzymes to the corresponding enzymes encoded by the genes in each strain is stated below each gene. (**b**) Comparison of the organization of the different γ-butyrolactone gene clusters of Rhodococci. A cluster is present in virtually all studied Rhodococci, with a highly similar organization. Only *R*. *opacus* R7 contains γ-butyrolactone clusters on its plasmids. GblA: γ-butyrolactone first biosynthetic enzyme. GblR: γ-butyrolactone receptor protein. GblE: γ-butyrolactone biosynthetic enzyme E, predicted to be a NAD-epimerase/dehydratase. GblD: γ-butyrolactone biosynthetic enzyme D, short chain dehydrogenase. GblC: γ-butyrolactone biosynthetic enzyme C, reductase.

Analysis of the predicted γ-butyrolactone gene cluster of *R*. *jostii* RHA1^7,11^ revealed the presence of a *gblA* gene (RHA1_RS22510) (Fig. 3a), encoding for GblA, the putative first enzyme in the biosynthetic pathway of γ-butyrolactones. It contains two AfsA repeats (Pfam03756 domain)^15^, the predicted active sites of GblA enzymes (see Fig. S2). A BLAST search with *gblA* in the *R*. *jostii* RHA1 genome sequence showed that this strain shares only a single copy. GblA_jostii_ has 37–41% amino acid (AA) identity with (partially) characterized homologues of *S*. *venezuelae* (JadW1), *S*. *coelicolor* (ScbA) and *S*. *griseus* (AfsA) (Fig. 3a). The homologues of these three *Streptomyces* species have 43–65% AA identity between each other. The **G**amma-**b**utyro**l**actone **R**eceptor protein (GblR) from *R*. *jostii* RHA1, annotated as a TetR regulator, has 34–36% AA identity with the corresponding proteins in the three *Streptomyces* strains (Fig. 3a); the homologues of the three *Streptomyces* species have 37–56% AA identity between each other. The γ-butyrolactone gene cluster of *R*. *jostii* RHA1 also includes a gene encoding a GblE enzyme (**G**amma-**b**utyro**l**actone biosynthesis **E**pimerase) with a NAD-epimerase/dehydratase predicted function. A (partially) characterized homologue is JadW2 of *S*. *venezuelae* (35% AA identity), shown to be essential for the synthesis of γ-butyrolactones^34^. However, it is not clear in which step of the biosynthesis pathway this enzyme acts. BLAST searches were also performed with GblD of *Streptomyces coelicolor*, a short chain dehydrogenase known to contribute to synthesis of γ-butyrolactones in some *Streptomyces* species, e.g. in *S*. *coelicolor* ^27^ and *S*. *venezuelae* ^17^. This yielded a large number of homologues with 30–40% AA identity to the query, encoded throughout the *R*. *jostii* RHA1 genome, and with a few of them located in the proximity of the *gbl* gene cluster. Also, two homologues of GblC were found encoded in the *R*. *jostii* RHA1 genome, with ~35% AA identity to the *S*. *coelicolor* GblC. These genes are not located in close proximity to the *gbl* gene cluster. Genes flanking the GBL gene cluster are not conserved between species.

All studied *Rhodococcus* strains possess a single predicted γ-butyrolactone gene cluster, except for *R*. *pyridinovorans*. *R*. *opacus* PD630 and *R*. *opacus* R7, which contain multiple γ-butyrolactone gene clusters in their genomes (Fig. 3b). *R*. *opacus* R7 contains γ-butyrolactone clusters on 2 different plasmids while in all other cases the clusters are located on the chromosome only. Most γ-butyrolactone gene clusters have a similar organization, with the *gblR* gene flanked by *gblA* and *gblE* but divergently oriented. The *gblE* gene however is not always present (Fig. 3b). We carefully checked whether other genes surrounding the predicted γ-butyrolactone gene clusters (Fig. 3) in the different *Rhodococcus* strains are conserved, but this was not the case.

### γ-Butyrolactones from *R*. *jostii* RHA1

**Figure 4:**
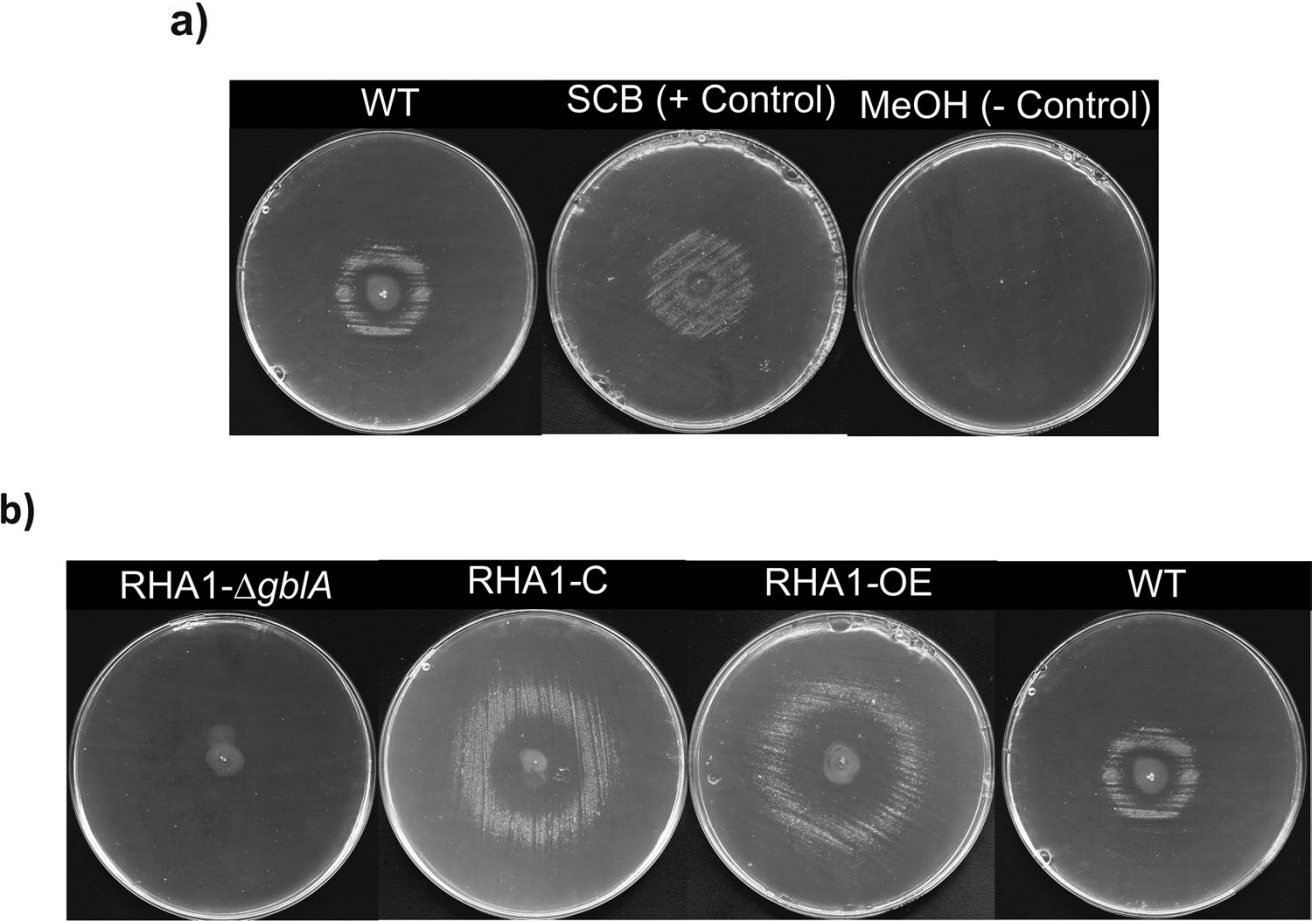
Detection of γ-butyrolactone(-like) molecules in *R*. *jostii* RHA1 ethyl acetate agar culture extracts by a GBL-specific reporter assay. When γ-butyrolactones are present in the sample the reporter strain *S*. *coelicolor* LW16/pTE134 forms a halo of growth around the sample application area in the centre of Km agar plates. Size of the halo of growth is indicative for the concentration of the diffusible γ-butyrolactones in the sample^33^. An excess of γ-butyrolactones inhibits the growth of the strain and a too low concentration cannot induce the expression of the Km resistance gene. (**a**) *R*. *jostii* RHA1 ethyl acetate extract (left), positive control with chemically synthesized 6-dehydro SCB2 (middle), negative control (solvent) (right). (**b**) *R*. *jostii*-Δ*gblA* (left), complemented strain (RHA1-C), *gblA* overexpression strain (RHA1-OE) and wild type strain (right). WT: wild type; MeOH: methanol

To analyse whether *R*. *jostii* RHA1 is producing any γ-butyrolactone(-like) molecules, a GBL-specific reporter assay developed for *S*. *coelicolor* (Km assay) was performed with ethyl acetate extracts of *R*. *jostii* RHA1 agar plates^33^. This test is based on release of the repression by the γ-butyrolactone receptor ScbR of transcription of a Km resistance gene in the *S*. *coelicolor* LW16/pTE134 indicator strain^25^. When plating this reporter strain on solid media with Km, it will only be able to grow if γ-butyrolactone molecules are present that are able to bind to the ScbR receptor protein and thereby allow transcription of the Km resistance gene. The *R*. *jostii* RHA1 extracts obtained, as described in the methods section, indeed induced the growth of the LW16/pTE134 reporter strain (Fig. 4a). *R*. *jostii* RHA1 thus indeed synthesizes γ-butyrolactone(-like) molecules (*Rhodococcus Jostii* Butyrolactone, RJB). This Km bioassay is very specific, since changes in the γ-butyrolactone aliphatic side chain are known to significantly affect the affinity of ScbR for these molecules^25^. Generic γ-butyrolactones also are not able to trigger this system^35^. *R*. *jostii* RHA1 apparently synthesizes one or more RJB molecules that are able to bind to the *S*. *coelicolor* γ-butyrolactone receptor protein ScbR, enabling growth of the reporter strain. In view of the high specificity of this assay it is likely that these *R*. *jostii* RHA1 RJB molecules are structurally most similar to SCBs, the γ-butyrolactone molecules of *S*. *coelicolor* ^23,25^.

To further analyse the γ-butyrolactone synthesis of *R*. *jostii* RHA1 the putative γ-butyrolactone biosynthesis gene *gblA* was deleted from the genome using unmarked gene deletion mutagenesis as described in Van der Geize *et al*.^29^. Ethyl acetate extracts of the deletion strain RHA1-Δ*gblA* were made and tested for RJB synthesis. In contrast to the wild type *R*. *jostii* RHA1, extracts of RHA1-Δ*gblA* did not induce the growth of the LW16/pTE134 reporter strain in the GBL-specific reporter assay, indicating the absence of molecules structurally similar to γ-butyrolactones capable of binding to ScbR (Fig. 4b). No phenotypical differences in growth rate, colony shape, pigment or antibiotic production, or cell shape under the microscope, were observed for this strain compared to the WT strain. In order to verify that the observed phenotype was caused only by the deletion of *gblA*, the RHA1-Δ*gblA* deletion strain was transformed with the vector pRM4*-gblA* containing *gblA*_jostii_ under the control of a constitutive strong promoter (ermE*) and the Cφ31 phage integrase. This strain showed production of γ-butyrolactones as detected by a GBL-specific reporter assay (Fig. 4b). pRM reintroduction of the wild type *gblA* gene resulted in a bigger halo of growth for the complemented strain than for the wild type strain indicating a higher production of γ-butyrolactones, probably due to the strong promoter used for the complementation (see Fig. 4b). In order to study the role of GblA in RJB production an overexpression strain (RHA1-OE) was constructed introducing pRM-*gblA* into wild type *R*. *jostii* RHA1. Next, RJB production by the RHA1-OE strain was analysed. Also in this case the Km bioassay showed a bigger halo of growth of the reporter strain compared to wild type *R*. *jostii* RHA1 extracts, which indicates a higher RJB concentration in the RHA1-OE sample due to a further diffusion from the application point. Thus, these experiments indicate that the overexpression of *gblA* results in an enhanced RJB production (Fig. 4b).

### Characterization of the γ-butyrolactone(-like) molecules synthesized by *R*. *jostii* RHA1

**Figure 5:**
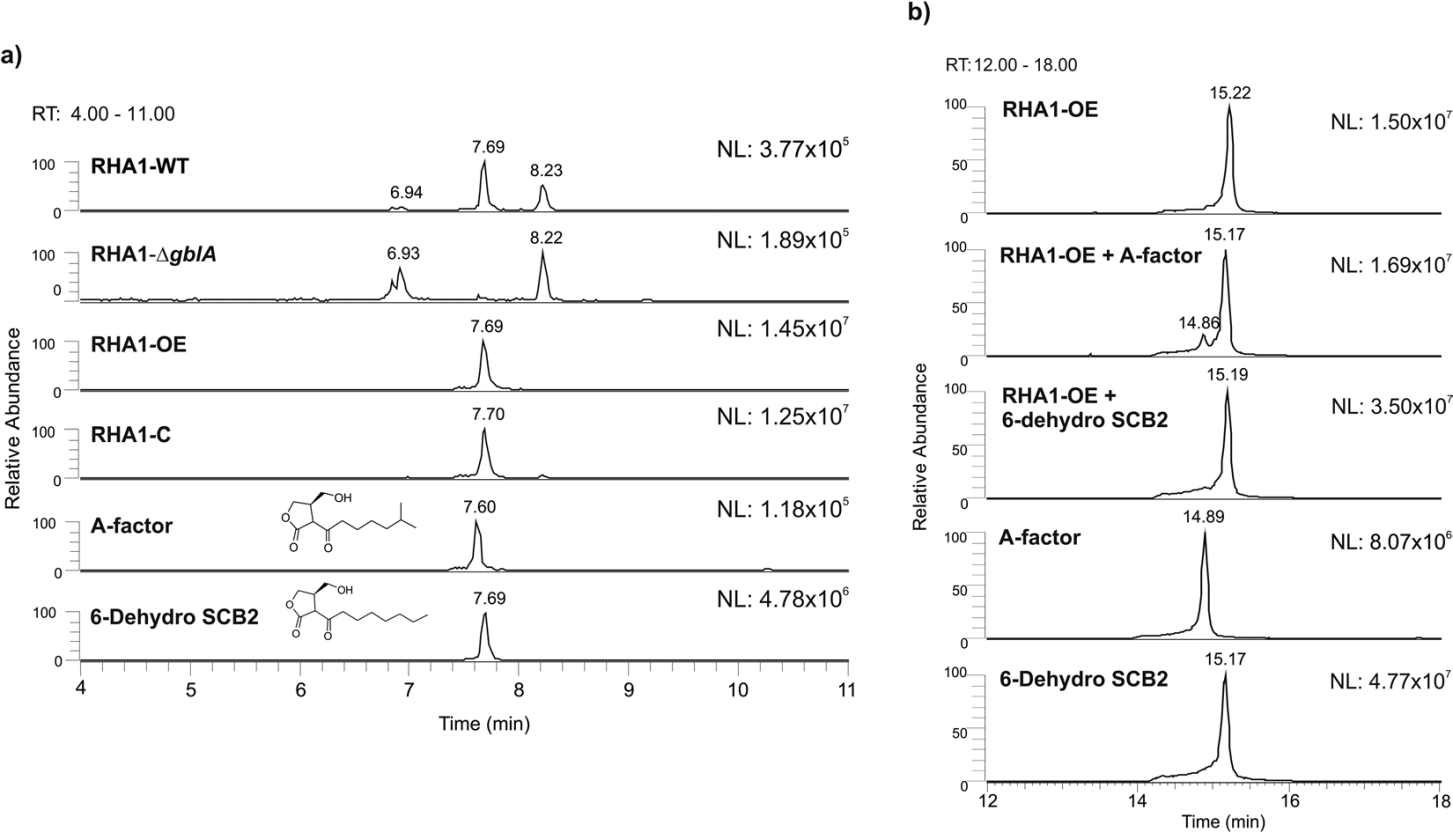
LC-MS analysis of ethyl acetate extracts of the various *R*. *jostii* RHA1 strains grown for 4 days on SMMS. (**a**) A peak eluting at 7.70 min with a mass of mass m/z 241.1441 amu [M-H]^−^ was detected in all samples except in RHA1-Δ*gblA*. RHA1-C, the complemented strain, and RHA1-OE, the *gblA* overexpression strain, both showed a higher intensity of this peak. This mass corresponds to the described γ-butyrolactone from *S*. *griseus* (A-factor) and the stereoisomer 6-dehydro SCB2, known to be an intermediate in the synthesis of the γ-butyrolactone SCB2 in *S*. *coelicolor*. The standard of A-factor showed a peak eluting at 7.60 min while the standard of 6-dehydro SCB2 eluted at 7.69 min. (**b**) Extracts of RHA1-OE spiked with standards of A-factor or 6-dehydro SCB2 at 50 ng/µl using a longer gradient to separate both peaks further. The spiked extract of *R*. *jostii* RHA1 with both standards confirmed that the molecule synthesized by *R*. *jostii* RHA1 has the same retention time and mass as 6-dehydro SCB2. (NL: Normalization Level; RT: Retention Time).

The extracts of wild type *R*. *jostii* RHA1, RHA1-Δ*gblA*, RHA1-OE and RHA1-C were analysed for RJBs by liquid chromatography coupled to a mass spectrometer (LC-MS). The structures of RJBs are apparently rather similar to those of the *S*. *coelicolor* γ-butyrolactones since they bind to ScbR, as evident from the Km bioassay (see above). Therefore, the LC-MS data was analysed searching for metabolites with masses similar to those of described for *S*. *coelicolor* γ-butyrolactones^23,25,36^. A peak eluting at 7.70 min with a mass of m/z 241.1441 amu [M-H]^−^ was detected in the extracts from *R*. *jostii* RHA1 wild type strain, RHA1-OE and RHA1-C, but it was missing in RHA1-Δ*gblA*. The *gblA* gene thus is essential for its synthesis, indicating that this peak indeed represents a *R*. *jostii* RHA1 RJB molecule (Fig. 5a). The mass of the detected *R*. *jostii* RHA1 RJB molecule corresponds to the A-factor signalling molecule of *S*. *griseus* and also to the intermediate compound 6-dehydro SCB2 of *S*. *coelicolor* ^27^ (Figs 2, 5a). Synthetic standards of A-factor and 6-dehydro SCB2 were also analysed on LC-MS, which eluted at 7.60 and 7.69 min, respectively (Fig. 5a) and with an exact mass of 241.1444 amu [M-H]^−^. The extracts of the different *Rhodococcus* strains yielded peaks with the same retention time as 6-dehydro SCB2. The RHA1-OE and RHA1-C peaks had a higher intensity than in the *R*. *jostii* RHA1 wild type strain, corresponding to the Km bioassay results, showing a bigger halo than seen with the wild type strain (Fig. 4b). To analyse whether the molecule detected in the *R*. *jostii* RHA1 extracts is similar to 6-dehydro SCB2 or to A-factor, the extract from RHA1-OE was spiked with the synthetic standards of these compounds at 50 ng/µl and run in the LC-MS with a longer gradient (Fig. 5b). As a control, a mixture of both standards (A-factor and 6-dehydro SCB2) was also run in the same conditions. The mixture of both standards and the extract spiked with the standard of A-factor showed two different peaks at 14.86 min and 15.17 min, corresponding to A-factor and 6-dehydro SCB2, respectively. When the extract was spiked with 6-dehydro SCB2 the peaks completely overlapped, confirming that the single RJB detected is structurally identical to 6-dehydro SCB2 (Fig. 5b). The *R*. *jostii* RHA1 samples were also compared to the available chemically synthesized standards of *S*. *coelicolor* γ-butyrolactones^27^, but we were not able to find any other known γ-butyrolactone(-like) molecules in *R*. *jostii* RHA1 extracts.

When the samples were screened for masses between 187 and 350, a mass range that includes all described γ-butyrolactones, two peaks had a higher intensity in the RHA1-OE and RHA1-C strains than in the *R*. *jostii* RHA1 wild type; these two peaks were not visible in the RHA1-Δ*gblA* deletion strain (see Fig. S3). One peak eluted at 7.08 min and showed three different masses, m/z 211.0972 amu [M-H]^−^, m/z 279.1369 amu [M-H]^−^ and m/z 289.1658 amu [M-H]^−^. Another peak eluted at 7.88 min that corresponds to a mass of m/z 255.1236 amu [M-H]^−^. None of these masses correspond to known γ-butyrolactones, including the recently described ones in Sidda *et al*.^36^ or Xu *et al*.^37^.

The absence of a halo in the GBL-specific reporter assay in the *gblA* deletion mutant, together with LC-MS analysis of the extracts of the *gblA* deletion mutant, are clear evidence that (no detectable amount of) GBL is not produced anymore by the deletion mutant and confirm the role of GblA in biosynthesis of this molecule.

### Phenotypical characterization of constructed *R*. *jostii gblA* strains

**Figure 6:**
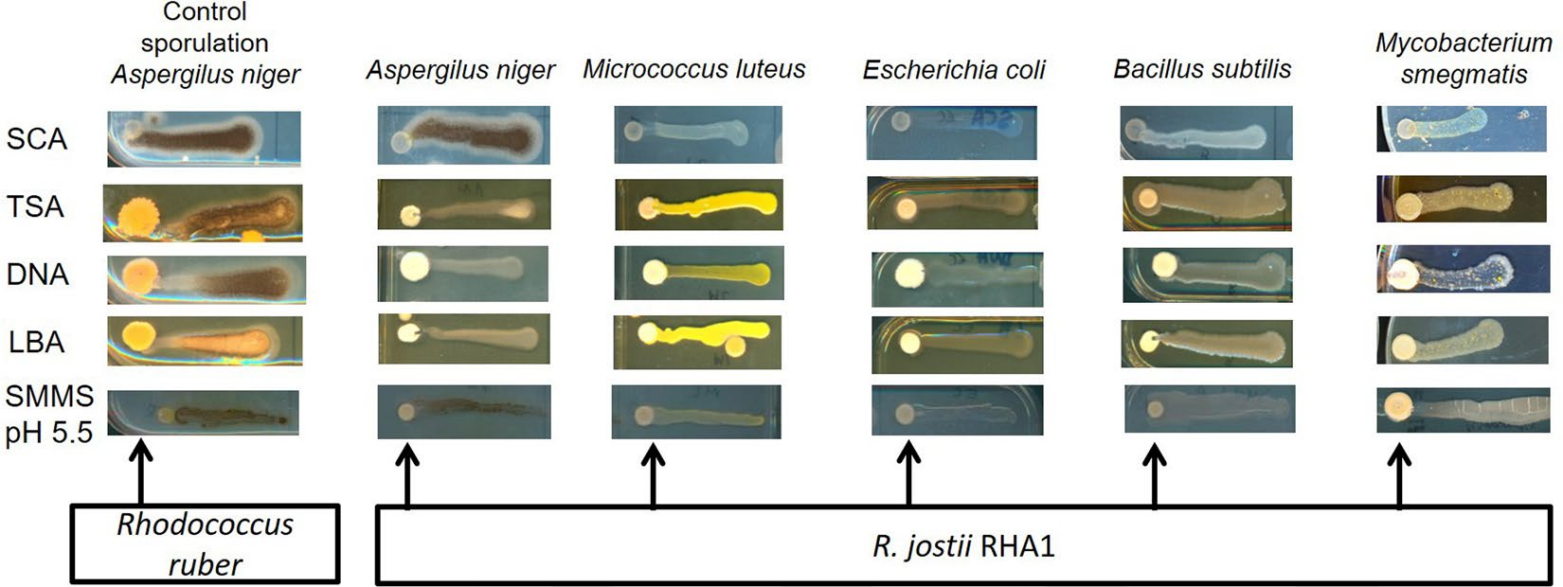
Bioactivity tests with *R*. *jostii* RHA1 spotted directly from glycerol stocks onto SCA, TSA, DNA, LBA and SMMS (pH 5.5) agar plates using *A*. *niger*, *M*. *luteus*, *E*. *coli*, *B*. *subtilis* and *M*. *smegmatis* as antimicrobial bioassay strains. After 4 days of incubation, the antimicrobial bioassay strains were applied on a horizontal line towards the *R*. *jostii* RHA1 patch. Inhibition of *A*. *niger* sporulation was observed on TSA, DNA and LBA (note loss of black pigment from the conidia compared to the control showing *A*. *niger* sporulation when plated next to *Rhodococcus ruber*). Growth inhibition of *M*. *luteus* and *M*. *smegmatis* was observed on SCA and SMMS, respectively (especially visible close to the *R*. *jostii* RHA1 patch at the left).

The γ-butyrolactone system is known to regulate secondary metabolite synthesis, morphogenesis or both, in Streptomycetes^15,38^. *R*. *jostii* RHA1 has almost 120 putative secondary metabolite biosynthetic gene clusters in its genome and most of them are uncharacterized^4^. Wild type *R*. *jostii* RHA1 was screened for secondary metabolite production during growth on different agar media. Production of bioactive compounds was tested with various indicator strains, two Gram-positive strains (*Micrococcus luteus* and *Bacillus subtilis*), one acid-resistant Gram-positive strain (*Mycobacterium smegmatis*), a Gram-negative strain (*Escherichia coli*) and a fungal species (*Aspergillus niger*). These antimicrobial bioassay strains were plated next to the *Rhodococcus* colonies. *R*. *jostii* RHA1 WT exerted clear inhibition of growth towards *M*. *luteus* on SCA medium and *M*. *smegmatis* in low pH SMMS (Fig. 6). Inhibition of sporulation of *A*. *niger* was observed on LBA, TSA and DNA agar media (Fig. 6).

The RHA1-Δ*gblA* deletion strain and the RHA1-OE overexpression strain were also analysed for changes in antimicrobial production in different growth media. No difference in bioactivity was observed between *R*. *jostii* RHA1 wild type and derived strains in any medium or with any antimicrobial bioassay strain tested. The growth on plate and colony shapes of all constructed strains were also analysed on all tested solid media, and their cell shapes in liquid LB medium, however, no differences were observed compared to the wild type strain (data not shown).

### Interaction between *R*. *jostii* RHA1 and *S*. *coelicolor* M145

**Figure 7:**
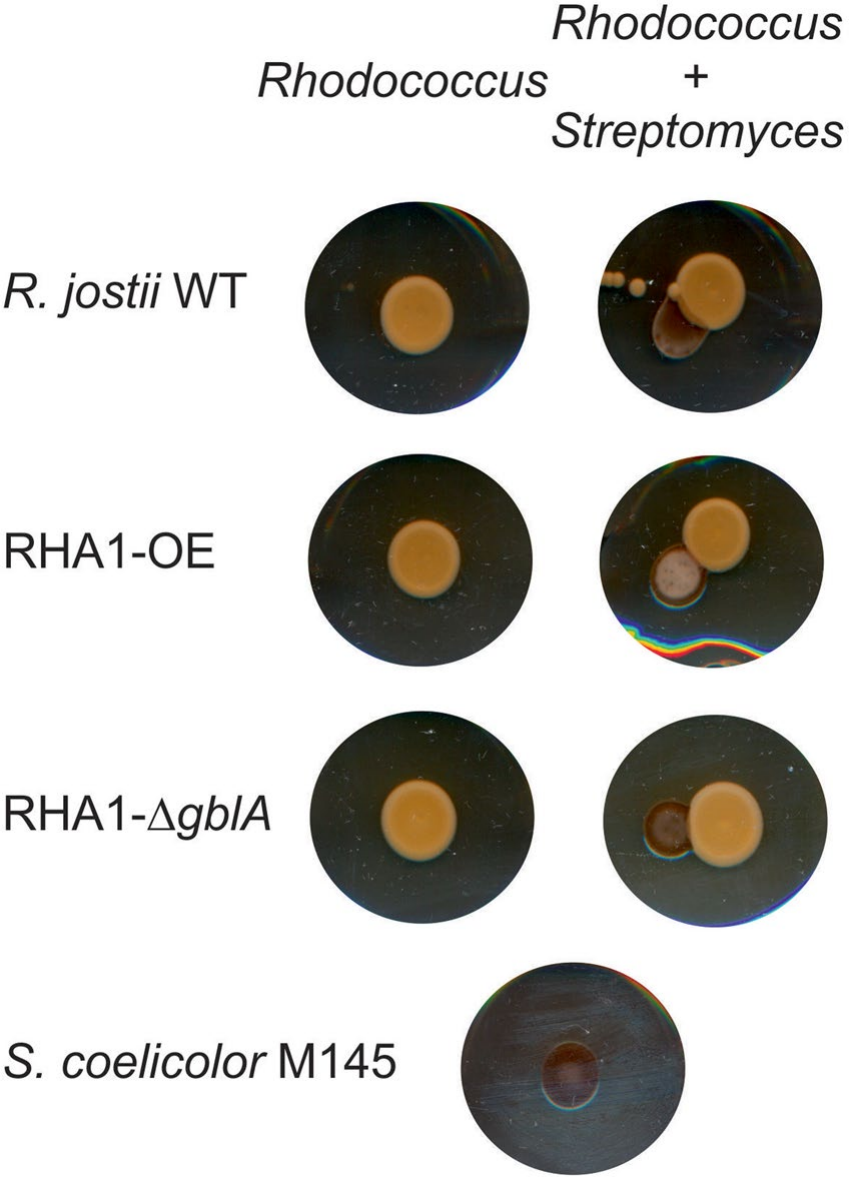
Interactions observed between *R*. *jostii* RHA1 WT, RHA1-OE and RHA1-Δ*gblA* with *S*. *coelicolor* M145 on MSM agar containing casamino acids. *Rhodococcus* strains were spotted directly from glycerol stocks and grown for 4 days before spores of *S*. *coelicolor* M145 were plated next to the patches of the *Rhodococcus* strains. On the left, the different strains from *R*. *jostii* RHA1 were grown separately. On the right, *S*. *coelicolor* M145 was grown next to the different *R*. *jostii* RHA1 strains. At the bottom*, S*. *coelicolor* M145 grown on its own.

The GBL-specific reporter assay performed with extracts from *R*. *jostii* RHA1 indicate that the *R*. *jostii* RHA1 RJB interacts with the γ-butyrolactone receptor protein ScbR of *S*. *coelicolor* (see above). These different genera thus may be capable of interspecies communication. To study a possible interaction between *R*. *jostii* RHA1 and *S*. *coelicolor* M145, both strains were inoculated next to each other on agar plates. γ-Butyrolactones diffuse into the agar and therefore an exchange of signalling molecules between species is possible. *R*. *jostii* RHA1 was allowed to grow for 4 days, using carotene production (orange pigmentation) as indication that secondary metabolism was active. Subsequently, *S*. *coelicolor* M145 was plated next to it. As a control, *S*. *coelicolor* M145 and *R*. *jostii* RHA1 also were plated separately on the same agar media. After a further 24 h, *S*. *coelicolor* M145 developed aerial mycelium with its characteristic white pigmentation when grown next to the RHA1-OE overexpression strain, but not when growing next to the RHA1-WT and the RHA1-Δ*gblA* strains (Fig. 7).

Deletion of *S*. *coelicolor gblA* (*scbA*) did not affect the morphological development^12^. Effects of γ-butyrolactones on development of S. *coelicolor* was suggested in Kawabuchi *et al*.^39^, but they were unable to prove that this effect was due to γ-butyrolactones. This difference in sporulation (Fig. 7) may be due to the *R*. *jostii* RHA1 RJB alone, or caused by unknown RHA1-OE compounds accumulating in response to the enhanced synthesis of RJB.

## Discussion

Rhodococci are Gram-positive soil bacteria known for the great variety of catabolic pathways which are encoded in their relatively large chromosomes, 9.6 Mb in case of *R*. *jostii* RHA1^4^. Genome analyses showed that Rhodococci also contain a large number of uncharacterized putative secondary metabolite gene clusters^4^. Here we show that γ-butyrolactone gene clusters are not only present in the genomes of *R*. *jostii* RHA1*, R*. *equi* 103S^4,7,10^ *, R*. *opacus* B4 and *R*. *erythropolis* PR4^7^, but occur even more widespread in Rhodococci (Fig. 3). Analysis of the γ-butyrolactone gene cluster in *R*. *jostii* RHA1 predicted the presence of genes encoding various homologues of enzymes known to be involved in the γ-butyrolactone biosynthesis in *Streptomyces* ^15^, namely GblA, GblE and GblR. *R*. *jostii* RHA1 lacks the GblD enzyme, which is also not present in *S*. *griseus* (Fig. 2), suggesting that *R*. *jostii RHA1* may employ a similar biosynthetic pathway as *S*. *griseus*. However, the GblE enzyme encoded in *R*. *jostii* RHA1 is not present in *S*. *griseus*. Instead, this GblE enzyme is homologous to the γ-butyrolactone enzyme JadW2 from *S*. *venezuelae*, which is known to be essential for the production of γ-butyrolactones in this strain^34^ but it has an unknown biosynthetic role. In order to analyse whether there are more homologues to the γ-butyrolactone biosynthesis enzymes described in *Streptomyces* BLAST searches with *gblD* and *gblC* of *S*. *coelicolor* were performed. A large number of dehydrogenases with 30%-40% AA identity to GblD, were found spread throughout the *R*. *jostii* RHA1 genome. Also, two homologues of GblC were found encoded in the *R*. *jostii* RHA1 genome, with ~ 35% AA identity to the *S*. *coelicolor* GblC. Homologues of these enzymes with higher identity than the ones found in *R*. *jostii* RHA1 are also present in the genomes of different *Streptomyces* strains. These enzymes have never been reported to be part of the γ-butyrolactone biosynthesis pathways in these streptomycetes. Clearly, simple sequence analysis is not sufficient to predict the involvement of *R*. *jostii* RHA1 genes encoding homologous enzymes in the synthesis of RJB. Deletion mutagenesis of these putative *gblC*, *gblD* and *gblE* genes in *R*. *jostii* RHA1 followed by LC-MS analysis of cell extracts of these transformants strains, searching for intermediates accumulating, may serve to elucidate the biosynthetic pathway in this strain. Since this pathway has not been completely elucidated in *Streptomyces*, other not yet identified pathway specific enzymes may also be involved in the synthesis of these signalling molecules, but we were unable to identify them.

Our data provide the first evidence of γ-butyrolactone synthesis in the genus *Rhodococcus*. These molecules were proven to be able to bind to the γ-butyrolactone receptor protein from *S*. *coelicolor*. The binding of exogenous molecules to γ-butyrolactone receptor proteins from *Streptomyces* previously has been observed for extracts of the cultures of other non-*Streptomyces* species^38,40^. The latter study also suggested that *Amycolatopsis mediterranei and Micromonospora echinospora* produce an IM-2 type molecule and *Actinoplanes teichomyceticus* a VB type of γ-butyrolactone^40^. This conclusion was based on the efficiency by which these molecules bind to the *S*. *virginiae* and *S*. *lavendulae* γ-butyrolactone receptor proteins respectively, but no structural analysis has been performed. We used LC-MS analysis to compare the compounds in *R*. *jostii* RHA1 ethyl acetate extracts from solid media with different chemically synthesized standards of known γ-butyrolactones. We were unable to extract g-butyrolactones in high enough concentrations from liquid media for their detection, not even after 40 h of growth (data not shown). It thus appears that the system is not induced at all, or at least not strong enough, in liquid growth media. The *R*. *jostii* RHA1 RJB was identified as 6-dehydro SCB2 (Fig. 5). 6-dehydro SCB2 is an isomer of the γ-butyrolactone described in *S*. *griseus* (A-factor) and is a predicted precursor of one of the described γ-butyrolactones in *S*. *coelicolor* (SCB2)^27^. In *S*. *coelicolor*, a GblD enzyme reduces the keto group in carbon 6 to a hydroxyl group (Fig. 2). Many genes encoding enzymes with low similarity to GblD of *S*. *coelicolor* were found spread throughout the genome of *R*. *jostii* RHA1. However, we did not find a *gblD* homologue in the *R*. *jostii* RHA1 gene cluster (Fig. 3a), which corresponds to the observation that it is producing a 6-dehydro form of the molecule. When the samples were screened by LC-MS for a mass range that includes all known γ-butyrolactones, two peaks were observed in RHA1-OE and RHA1-C that were not present in the deletion strain and were in a lower intensity in the WT strain. The masses corresponding to these peaks did not match to those of any γ-butyrolactone described to date. These molecules could be γ-butyrolactones with novel structures, or totally different compounds, e.g. products of a biosynthesis pathway regulated by RJB. The detected mass of m/z 255.1236 amu [M-H]^−^ differs in m/z 14 from 6-dehydro SCB2. In further work we will attempt the isolation of sufficient amounts of these molecules for NMR analysis to elucidate their structures.

Unmarked deletion mutagenesis of the *gblA* gene in *R*. *jostii* RHA1 abolished RJB synthesis. Various homologues of this gene in *Streptomyces* species are known to be essential for biosynthesis of γ-butyrolactone molecules, catalyzing the first step of the biosynthesis, the condensation of a glycerol derivative with a fatty acid derivative (Fig. 2). Both Km bioassays (Fig. 4) and LC-MS analysis of extracts of the various *R*. *jostii* RHA1 (mutant) strains (Fig. 5) confirmed that *gblA* is essen2yrolactones are known to regulate secondary metabolism and/or morphogenesis in the genus *Streptomyces* ^15,38^. *R*. *jostii* RHA1 contains a large number of putative secondary metabolite clusters that are mostly uncharacterized. RJB may be involved in control of the expression of one or more of these clusters. Although a few *Rhodococcus* antimicrobials are known^41–44^ this genus has remained largely unexplored for production of secondary metabolites. In this work, we detected bioactivity of *R*. *jostii* RHA1 against *Micrococcus luteus, Aspergillus niger* and *Mycobacterium smegmatis*. Lariatins, cyclic peptides that have bioactivity against *Mycobacterium* species, were found in *R*. *jostii* K01-B0171^42^, but the enzymes involved in the synthesis of these compounds are not encoded in the genome from *R*. *jostii* RHA1. We have not been able to find a difference in antibiotic production, growth rate or colony shape between the *R*. *jostii* RHA1 WT, RHA1-Δ*gblA* and RHA1-OE strains, therefore further experiments are needed to analyse the role of γ-butyrolactone system in *R*. *jostii* RHA1. Mutagenesis analysis of GblR may help identify any *R*. *jostii* RHA1 genes that are regulated by its RJB. Various systems may be controlled by RJB in Rhodococci, analogous to the situation in the genus *Streptomyces*. In some species of *Streptomyces* γ-butyrolactones are known to be involved in morphogenesis and sporulation, as is the case in *S*. *griseus*. Deletion of *afsA* in *S*. *griseus* blocked its sporulation and streptomycin production^38,45^. The RJB in *R*. *jostii* RHA1 might be controlling the synthesis of one or more secondary metabolites that have remained unidentified, or it may be directly or indirectly influencing the primary metabolism in this strain.

The γ-butyrolactone system is known to be present in several Actinomycete genera^21,25,33,40^. γ-Butyrolactone molecules described in *Streptomyces* have differences in structure depending on the producing species. An exception is a γ-butyrolactone produced by *S*. *venezuelae* which was found to be identical to SCB3 from *S*. *coelicolor*. Actinomycetes are soil bacteria that live in a rich community of microorganisms. The γ-butyrolactone system may have developed as a way to communicate between different species^19,46^. To test whether such interspecies communication occurs between *R*. *jostii* RHA1 and *S*. *coelicolor*, we plated these strains next to each other. *S*. *coelicolor* sporulation clearly was accelerated when growing next to RHA1-OE compared to *S*. *coelicolor* growing alone or next to the RHA1 WT and RHA1-Δ*gblA* strains. These results indicate that compounds secreted by the *R*. *jostii* RHA1-OE strain affect morphological differentiation in *S*. *coelicolor*. This effect is known to be induced by γ-butyrolactone molecules in other *Streptomyces* species^15,47^ but has never been described before in *S*. *coelicolor*. The addition of 6-dehydro SCB2 to a confluent lawn of *S*. *coelicolor* however did not induce sporulation which indicated that the phenotypical difference observed is not a direct effect of this RJB. This phenomenon thus remains to be studied in more detail in future work.

This work reports on the synthesis of a γ-butyrolactone(-like) molecule by *R*. *jostii* RHA1. This RJB molecule appears to be structurally very similar to the γ-butyrolactones described in *Streptomyces* and interacts with the *S*. *coelicolor* butanolide system. In future work, we aim to elucidate the physiological roles of these signalling molecules in *Rhodococcus* metabolism, with specific interest in possible regulatory effects on representatives of the many secondary metabolite biosynthetic gene clusters in this genus. We have shown that *R*. *jostii* RHA1 produces compounds with antibiotic activity, with at least one of them active against *M*. *smegmatis* and therefore potentially also against the fast-emerging multidrug resistant *Mycobacterium tuberculosis*. Activation of cryptic secondary metabolite clusters in Rhodococci may potentially unlock the biosynthesis of novel compounds that are of interest to the pharmaceutical industry.

## Electronic supplementary material

Supplementary information
