# Supplementary material for: Molecular characterization of a Rhodococcus jostii RHA1 γ-butyrolactone(-like) signalling molecule and its main biosynthesis gene gblA
Source: Sci Rep. 2017 Dec 18;7:17743. doi: 10.1038/s41598-017-17853-6 (PMC5735094; doi:10.1038/s41598-017-17853-6)
Supplement: Supplementary file 1 — Supplementary information [file 41598_2017_17853_MOESM1_ESM.pdf]

Molecular characterization of a *Rhodococcus jostii* RHA1 γ-butyrolactone(-like) signalling molecule and its main biosynthesis gene *gblA*

Ana Ceniceros, Lubbert Dijkhuizen*, and Mirjan Petrusma

Supplementary information

Supplementary Table S1 Media components used in this study.

Media components per litre							
**Trypton Soya Agar**	**Difco Nutrient Agar**	**Luria Broth Agar**	**Starch Casein Agar**	**Minimum Salt Media +Casamino acids**	**MSM-Nitrogen deficient**	**SMMS**	**SMMS trace elements**
Tryptone 15 g	Beef extract 3 g	Tryptone 10.0 g	Starch 5 g	Glucose 15 g	Glucose 15 g	Bacto casamino acids, Technical 2 g	$ZnSO_4 \cdot 7H_2$ 0.1 g
Soytone-enzymatic digest of soybean meal 5 g	Peptone 5 g	Yeast extract 5.0 g	Casein 1 g	Na_2HPO_4. $2H_2O$ 3.57 g	$Na_2HPO_4.2H_2O$ 3.57 g	TES buffer 5.73 g	$FeSO_4 \cdot 7H_2O$ 0.1 g
Sodium chloride 5 g	Agar 15 g	Sodium chloride 5.0 g	Agar 20 g	$MgSO_4.H_2O$ 0.2 g	$MgSO_4.H_2O$ 0.2 g	2.46% $MgSO_4 \cdot 7H_2O$ 5 ml	$MnCl_2 \cdot 4H_2O$ 0.1 g
Agar 15 g		Agar 10.0 g	pH 7	$CaCO_3$ 0.02 g	$CaCO_3$ 0.02 g	Trace elements 2 ml	$CaCl_2 \cdot 2H_2O$ 0.07 g
				$FeSO_4.7H_2O$ 0.01 g	$FeSO_4.7H_2O$ 0.01 g	55% Glucose$\cdot H_2O$ 20 ml	NaCl 0.1 g
				H_3BO_3 50 µg	H_3BO_3 50 µg	0.69% $NaH_2PO_4 \cdot H_2O$ + 0.87% K_2HPO_4 5 ml	
				$CuSO_4.H_2O$ 10 µg	$CuSO_4$-H_2O 10 µg	Adjust pH to 7.2	
				$MnSO_4.4H_2O$ 40 µg	$MnSO_4.4H_2O$ 40 µg	Lab-M Agar 20 g	
				$ZnSO_4.7H_2O$ 40 µg	$ZnSO_4.7H_2O$ 40 µg		
				$Na_2MoO_4.2H_2O$ 24 µg	$Na_2MoO_4.2H_2O$ 24 µg		
				Bacto casamino acids Technical 2 g	Agar 20 g		
				Agar 20 g	pH 7.2		
				pH 7.2			

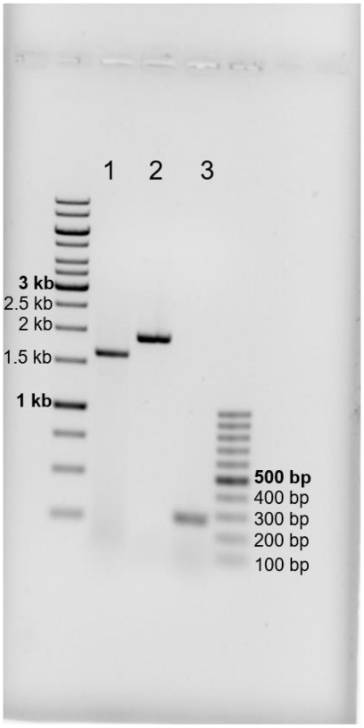

Expected size:
WT strain
1. 2585 bp afsA check Fw and Rv 1
2. 2326 bp afsA check Fw and Rv 2
3. 969 bp ScbA-jostii-NdeI-F and ScbA-jostii-BamHI-R

Deletion strain
1. 1883 bp afsA check 1 Fw and Rv
2. 1624 bp afsA check 2 Fw and Rv
3. 267 bp ScbA-jostii-NdeI-F and ScbA-jostii-BamHI-R

Supplementary Figure S1 PCR check of the *afsA* deletion strain. Expected size of the products for the wild type and the deletion strain is marked on the right. The obtained bands correspond to the deletion of *afsA*

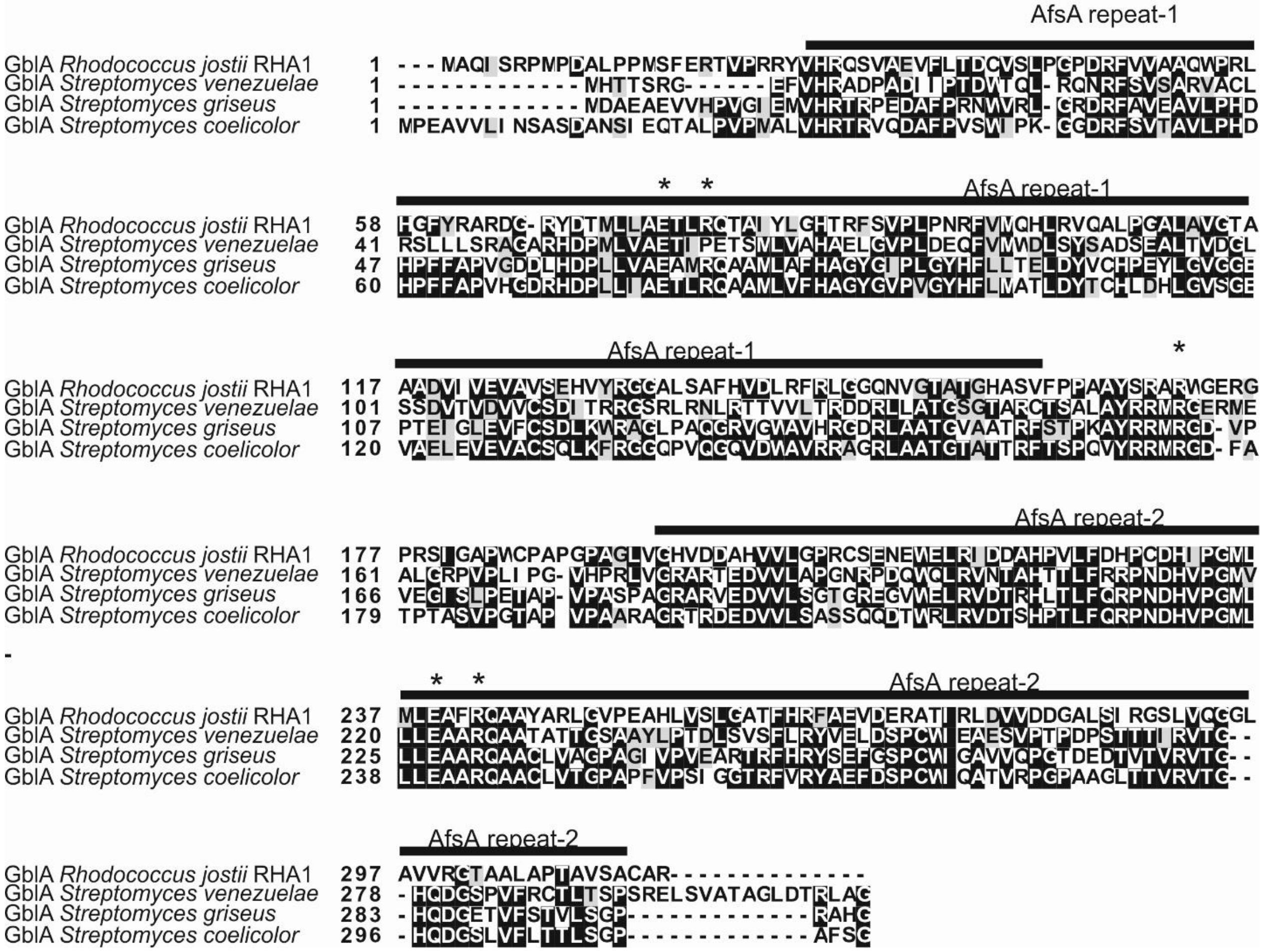

Supplementary Figure S2 Alignment of the predicted GblA enzyme from *R. jostii* RHA1 with the previously descrived GblA enzymes from *S. venezuelae, S. griseus* and *S. coelicolor*. AfsA repeats predicted by Uniprot and CCD are marked with a black line on the top. Active sites in *S. coelicolor* GblA are marked with a star[1, 2]

a)

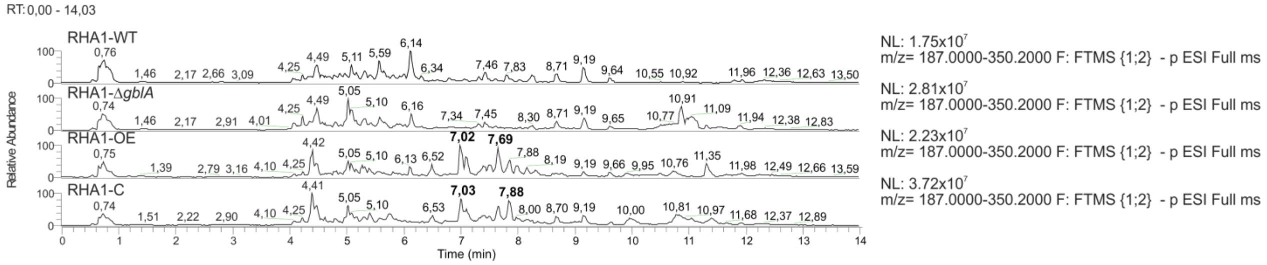

b)

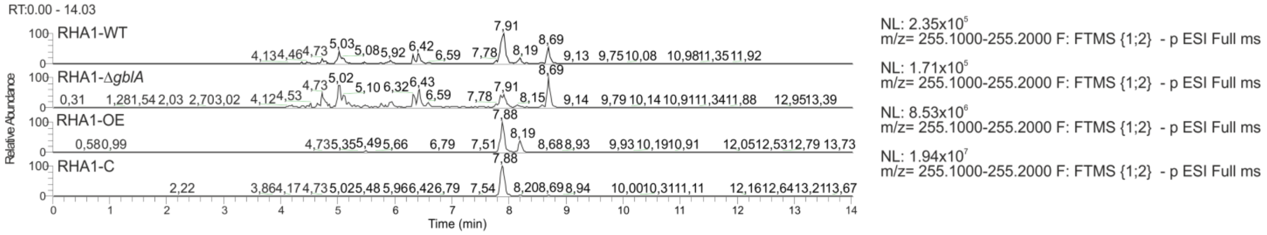

c)

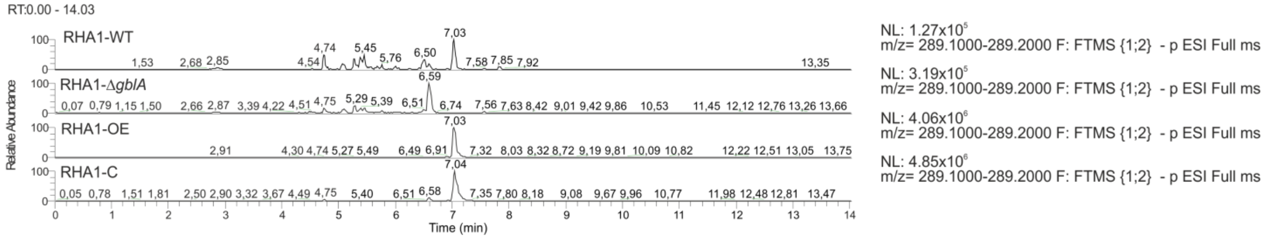

Supplementary Figure S3. LC-MS analysis of ethyl acetate extracts from different *R. jostii* RHA1 strains. a) LC-MS Chromatogram in the mass range of *m/z* 187-350 $[M-H]^-$. In bold, peaks found in extracts of the RHA1-OE and RHA1-C strains that were in a much lower intensity in the wild type and not observed in strain RHA1-Δ*gblA*. b) LC-MS chromatogram in the mass range *m/z* 255.1 and 255.2 $[M-H]^-$ to search for the mass found in the 7.88 min peak (m/z 255.1236 amu $[M-H]^-$). This peak was present in a higher intensity in the extracts of strains RHA1-OE and RHA1-C ($9.53x10^6$ and $1.94x10^7$ respectively) than in the wild type ($2.35x10^5$). A peak at 7.91 min was observed in the RHA1-Δ*gblA* strain but this peak corresponded to a different mass that was also found in the other strains tested. C) LC-MS chromatogram in the mass range *m/z* 289.1 and 289.2 $[M-H]^-$ searching for the mass m/z 289.1658 amu $[M-H]^-$, one of the major masses found in the 7.03 min peak. A peak was observed in all strains except RHA1-Δ*gblA*. As in the previous case, the intensity of this peak was higher in strains RHA1-OE and RHA1-C ($4.06x10^6$ and $4.85x10^6$ respectively) than in the RHA1-WT strain ($1.27x10^5$). None of these masses correspond to described γ-butyrolactones.

References

1. Martin-Sanchez, L. *et al*. Identification and characterisation of enzymes involved in γ-butyrolactone biosynthesis in *Streptomyces coelicolor.* In: Quorum sensing in *Streptomyces coelicolor*: Regulation of the SCB signalling system that controls the synthesis of antibiotics. *PhD thesis L. Martin-Sanchez, University of Groningen* **http://www.rug.nl/research/portal/files/26679124/Chapter_3.pdf**, 91-138 (2016).

2. Hsiao, N. H. *et al*. ScbA from Streptomyces coelicolor A3(2) has homology to fatty acid synthases and is able to synthesize gamma-butyrolactones. *Microbiology* **153**, 1394-1404 (2007).
